# *Paracoccidioides brasiliensis* infection promotes thymic disarrangement and premature egress of mature lymphocytes expressing prohibitive TCRs

**DOI:** 10.1186/s12879-016-1561-8

**Published:** 2016-05-17

**Authors:** Rosaria Di Gangi, Thiago Alves da Costa, Rodolfo Thomé, Gabriela Peron, Eva Burger, Liana Verinaud

**Affiliations:** Department of Structural and Functional Biology, Institute of Biology, State University of Campinas, Rua Monteiro Lobato, 255, Cidade Universitária, SP Brazil; Instituto de Ciências Biomédicas, Universidade Federal de Alfenas, Alfenas, MG Brazil

**Keywords:** Paracoccidioides brasiliensis, Paracoccidioidomycosis, Thymic atrophy

## Abstract

**Background:**

Paracoccidioidomycosis, a chronic granulomatous fungal disease caused by *Paracoccidioides brasiliensis* yeast cells affects mainly rural workers, albeit recently cases in immunosuppressed individuals has been reported. Protective immune response against *P. brasiliensis* is dependent on the activity of helper T cells especially IFN-γ-producing Th1 cells. It has been proposed that *Paracoccidioides brasiliensis* is able to modulate the immune response towards a permissive state and that the thymus plays a major role in it.

**Methods:**

In this paper, we show that acute infection of BALB/c mice with *P. brasiliensis* virulent isolate (Pb18) might cause alterations in the thymic environment as well as the prohibitive TCR-expressing T cells in the spleens.

**Results:**

After seven days of infection, we found yeast cells on the thymic stroma, the thymic epithelial cells (TEC) were altered regarding their spatial-orientation and inflammatory mediators gene expression was increased. Likewise, thymocytes (differentiating T cells) presented higher migratory ability in ex vivo experiments. Notwithstanding, *P. brasiliensis*-infected mice showed an increased frequency of prohibitive TCR-expressing T cells in the spleens, suggesting that the selection processes that occur in the thymus may be compromised during the acute infection.

**Conclusion:**

In this paper, for the first time, we show that acute infection with *Paracoccidioides brasiliensis* yeast cells promotes thymic alterations leading to a defective repertoire of peripheral T cells. The data presented here may represent new mechanisms by which *P. brasiliensis* subverts the immune response towards the chronic infection observed in humans.

**Electronic supplementary material:**

The online version of this article (doi:10.1186/s12879-016-1561-8) contains supplementary material, which is available to authorized users.

## Background

The thymus is a primary lymphoid organ, where the differentiation and maturation of T-cell takes place. A number of cells and soluble proteins compose the thymic microenvironment and are responsible for forming a three dimensional network that provides mechanical support and fundamental stimulus for T-cell development [1]. In this process, intra-thymic migration of T-cell precursors plays an essential role [2].

The T-cell development in the thymus initiates after the entrance of double-negative (DN) T lymphocyte precursors at the cortico-medullary junction. Signals from cortical Thymic Epithelial Cells (cTEC) and IL-7 induce the DN thymocytes to migrate to the sub-capsular region of the cortex and express the specific chemokine receptors CXCR4, CCR7 and CCR9 [3, 4]. Then the V (D) J rearrangement of T Cell Receptor (TCR) β chains occurs [5–7] and there is formation of pre-TCR complex, followed by the expression of CD4 and CD8 co-receptors [1, 8, 9].

The repertoire of the αβ polypeptide chains in TCR associated with CD3 provides the recognition of foreign antigens and the specificity of the αβTCR depends on the Vβ, the variable domain of the β chain [5]. Thus, double-positives (DP) thymocytes carrying functional TCRαβ interact with cTECs, receiving survival signals and maturating into single-positive (SP) thymocytes (9) which migrate though the cortex to the medullar region under the attraction of CCL19 and CCL21 chemokine ligands expressed by medullary (m) TECs, where they will undergo another repertoire screening called negative selection [10–13]. In this process the Autoimmune Regulator (Aire) gene plays a crucial role promoting the expression of tissue specific self-antigens within the thymus, if thymocytes express TCR segments that have high avidity for these self-antigens-MHC complex, they are eliminated by apoptosis [14].

Studies in BALB/c mices have shown that TCR segments Vβ5 and Vβ12 are normally eliminated in the process of negative selection, receiving the denomination of prohibited segments, numerous researches links them to damage to self events [15–18]. Finally, after the central tolerance processes the thymocytes become naïve T cells and migrate to secondary lymphoid organs. In fact, the thymus has a crucial rule in cell-mediated immunity that is the main host defense against intracellular pathogens, such as fungi.

The fungus *Paracoccidioides brasiliensis* (Pb) is the mainly etiological agent of Paracoccidioidomycosis (PCM) that is a severe chronic and systemic mycosis. This mycosis account with cases widely distributed throughout Central and South America countries [19]. Infection disseminate to other tissues, resulting in damages of the skin, mucous membranes, lungs, Central Nervous System (CNS), spleen and lymph nodes compromising their functions [20]. During infection, the host fails to protect itself against Pb [20–22]. Several mechanisms have been proposed for the impaired immune response, such as failure in antigen presentation, reduced T lymphocyte function and production of Th2-related cytokines like IL-4 and IL-10 [23, 24]. Interestingly, we found that experimental infection with the high virulent clinical isolate Pb18 is followed by thymic involution [20].

Thymic atrophy has been described in many infections and metabolic disturbances, such as malaria, HIV, diabetes and malnutrition [20, 25–29]. The integrity of the thymic microenvironment is important for the maturation of thymocytes, where changes in the thymic microenvironment may compromise the T cell repertoire in the periphery, leading to immunosuppression or autoimmune diseases.

In this sense, we have recently shown that thymic atrophy in experimental malaria predisposes mice to exacerbated Experimental Autoimmune Encephalomyelitis (EAE), the mouse model of Multiple Sclerosis [30].

It is not clear which events are involved in the thymic atrophy seen in Pb18 infection nor whether organ atrophy would result in altered T cell repertoire in the periphery. In this study, we aimed to investigate the alterations caused by *P. brasiliensis* infection in the function and structure of the thymus as well as the effects of these alterations in T cell repertoire from peripheral lymphoid organs. It was found that in the course of infection, the thymus is rendered atrophic by means of epithelial cell spatial disarrangement and increased gene expression of inflammatory mediators. Notwithstanding, infected mice showed an increase in the frequency of prohibitive TCR-expressing T cells in the periphery. These results shed light on possible additional mechanisms of immunosuppression caused by Pb infection.

## Methods

### Mice

Eight-week old male BALB/c mice were used in this study. Mice were allocated in specific-pathogen free condition in transparent acrylic plastic isolators (dimensions: 37, 9 cm × 19,7 cm × 12,7 cm, 5 animals per cage) with absorbent material (wood shavings) in ventilated racks (Alesco, SP, Brazil) on a 12 h light/dark cycle and controlled temperature environment (20°-24 °C). Sterile water and food (Nuvilab CR-1; Nuvilab, PR, Brazil) were provided ad libitum. Once a week the bedding of the isolators was changed with autoclaved wood shavings. This study was conducted according to the ethical principles of animal research adopted by the Brazilian National Council for the Control of Animal Experimentation (CONCEA) and was approved and carried out in accordance with the guidelines of the Institutional Committee for Use of Laboratory Animals (CEUA, protocol number # 2969–1). All procedures aforementioned are in accordance to the ARRIVE Guidelines for reporting animal research and the completed ARRIVE guidelines checklist is described in (Additional file 1: Supplemental data).

### Strain and infection

The virulent strain Pb18 of *P. brasiliensis* used in this study was maintained at 37 °C in its yeast form in Fava’s Netto medium and used at the seventh day of cells culture growth. The fungal virulence was maintained by followed recommendation [31–33]. The fungal mass was collected and suspended in sterile phosphate-buffered saline (PBS 0.01 M pH 7.4), mixed three times for 30 seconds with 60 seconds in between on a Vortex-mixer, centrifuged and then washed in PBS. Fungal viability was verified using trypan blue dye exclusion in a hemocytometer. Mice (*n* = 5 mice/group) were injected intraperitoneally with 5x10^6^ viable yeast cells contained in 0.2 mL of PBS or with PBS alone. Infection was carried out for seven days when mice were euthanized by deep anesthesia with ketamine/xylazine (100 mg/kg ketamine hydrochloride and 5 mg/kg xylazine hydrochloride), and the thymuses and spleens were excised. Groups of 5 mice were used for each experiment. BALB/c mice were used as they are the most common experimental model of infection with Pb, evaluated as intermediate in susceptibility patterns to PCM [34].

### Thymus weight and histological analysis

Whole thymuses were weighted individually from control and infected mice. For histological analysis, the thymuses were fixed in buffered 4 % paraformaldehyde followed by dehydration in ethanol, diaphanization in xylene and inclusion in Paraplast Plus (Sigma). Thin slices were made (5 μm) in microtome and stained with Hematoxylin and Eosin while some slices were submitted to Grocott staining by sliver impregnation of viable yeast cells. The slices were analyzed under bright light field microscope equipped with camera (Olympus, Japan).

### Analysis of thymic epithelial cells distribution

The thymuses were processed to cryotomy and slices of 5 μm were made in cryostat. Briefly, free reactive sites in the slices were blocked with PBS plus Bovine Serum Albumin (BSA 1 %), followed by immunostaining for cytokeratin 8 (TROMA-I, DHSB Hybridoma, USA) and cytokeratin 14 (ab53115, Rabbit polyclonal to Cytokeratin 14, Abcam, UK). After, Cy2- and Cy3-conjugated secondary antibodies were added. At the end of incubation period, the slices were mounted in Vectashield (Vector Laboratories, Inc. Burlingame, USA) and were analyzed under epifluorescence microscope (Olympus, Japan). Images were analyzed in Image J for integrated density. Eight slides from each group were analysed (*n* = 4).

### Thymocytes subpopulation

The thymuses were homogenized individually in staining buffer (PBS 0.02 M pH 7.2 enriched with 2 % Fetal Calf Serum) and cell number was estimated by hemocytometer. Flow cytometry staining was performed following a previously described protocol [35]. Briefly, one million cells were incubated with fluorochrome-conjugated monoclonal antibodies in a final volume of 100 μL for 20 min at 4 °C. Preparations were acquired with a Gallios flow cytometer (Beckman Coulter, USA) and data was analyzed using FlowJo VX (Tree Star Inc., Ashland, OR, USA). Cells were stained with anti-CD4 (clone H129.12 – BD Biosciences, CA, USA) and ant-CD8 (53–6.7–BD Biosciences, CA, USA).

### Real time PCR

Thymic mRNA was extracted individually using the Qiagen RNAeasy Mini Kit (MD, USA) according to the manufacturer’s instructions. Quantification was performed in Nanodrop2000 (Thermo) and the mRNA was reverse transcripted into cDNA using High Capacity Kit (Applied Biosystems) by following manufacturer’s instructions. Then the Taqman Gene Expression Master Mix (Applied Biosystems) was employed for real time PCR amplification using the Applied Biosystems 7500 Real-Time PCR Systems (CA, USA). Levels of mRNA expression were normalized to housekeeping gene GAPDH mRNA levels. The primers used in this study are: IL-2 (Mm00434256_m1), IL-7 (Mm01295803_m1), IL-10 (Mm00439614_m1), IL-17 (Mm00439618_m1), TNFα (Mm00443260_g1), AIRE (Mm00477461_m1), GAPDH (Mm99999915_g1). Expression levels of genes were represented as a relative copy numbers by using the method of delta threshold (2^-ΔΔCt^).

### Chemokine in the thymus of mice infected with P. brasiliensis

Total protein was extracted from infected and control mice using RIPA lysis buffer following manufacturer’s instructions (Millipore, CA, USA). Total protein concentration was measured by the method of BRADFORD following manufacturer’s instructions (Bio-Rad, CA, USA). The levels of CCL19 were measured by ELISA Kit (R&D Biosystems, MN, USA) followed by manufacturer’s instructions. To determine the expression of the CCR7 chemokine receptor in thymocytes, a flow cytometry analysis was performed in single cell suspensions originated from healthy and infected mice using CCR7 antibody (clone 4B12 – BD Biosciences, CA, USA) gated in CD45RA^+^ (clone 14.8–BD Biosciences, CA, USA) cells. The CCR7 expression was determined by Mean Fluorescence Intensity (MFI) parameter in FlowJo VX (Tree Star Inc., OR, USA).

### Cell migration assay

Migration assays were performed in 24 well plates containing inserts with 5 μm diameter pores according to the manufacturer’s instructions (Corning Life Sciences, MA, USA). The membranes were incubated with 10 μg/mL of BSA for 1 h at 37 °C and then blocked with PBS/BSA 0.5 % for 45 min at 37 °C. After blocking RPMI/BSA 1 % or RPMI/CCL19 (100 μg/mL; R&D Systems, MN, USA) were added to the lower chamber of each trans–well assay. Thymuses cellular suspension was obtained by forcing the organ through a 70 μm cell strainer and suspending in RPMI/BSA 1 %. Then 100 μL of cell suspension (containing 2.5×10^6^ cells) from control and infected mice, counted on a hemocytometer, were added to the upper chamber of each trans-well assay. Cells were allowed to migrate for 3 h at 37 °C and upon completion; cells were recovered from the lower chamber and counted on a hemocytometer. Percent migration was calculated from the ratio of cells in the lower chamber to the initial input, and multiplying by 100.

### Expression of prohibited TCRVβ5 and Vβ12 segments in spleens of mice infected with P. brasiliensis

The analysis of prohibited TCR-expressing T cells followed a previous recommendation [36]. Briefly, the spleens from control and infected mice were collected and homogenized individually in PBS plus fetal bovine serum 5 %. After red blood cells (RBC) lysis, the cellular concentration was adjusted and 1×10^6^ cells of each mouse were transferred to appropriate cytometer tubes. Cells were incubated with fluorochrome-conjugated monoclonal antibodies for 20 min at 4 °C. Events were acquired on a flow cytometer (Gallios, Beckman Coulter, CA, USA) and data was analyzed using FlowJo VX (Tree Star Inc., OR, USA). Antibodies used in this study were anti-CD4 (clone H129.12–BD Biosciences, CA, USA), CD8 (clone 53–6.7 – BD Biosciences, CA, USA), TCRVβ12 (clone MR9-4 eBioscience, CA, USA), TCRVβ5 (clone MR11-1 eBioscience, CA, USA).

### Statistical analysis

Statistical analyses were performed using GraphPad 6 software and Student’s *t*-*test* was used for 2-group comparisons. A P value of less than 0.05 was considered statistically significant. Values are expressed as means ± standard error mean (SEM).

## Results

### Acute Paracoccidioides brasiliensis infection leads to atrophy, architecture changes and fungal invasion of the thymus

**Fig. 1:**
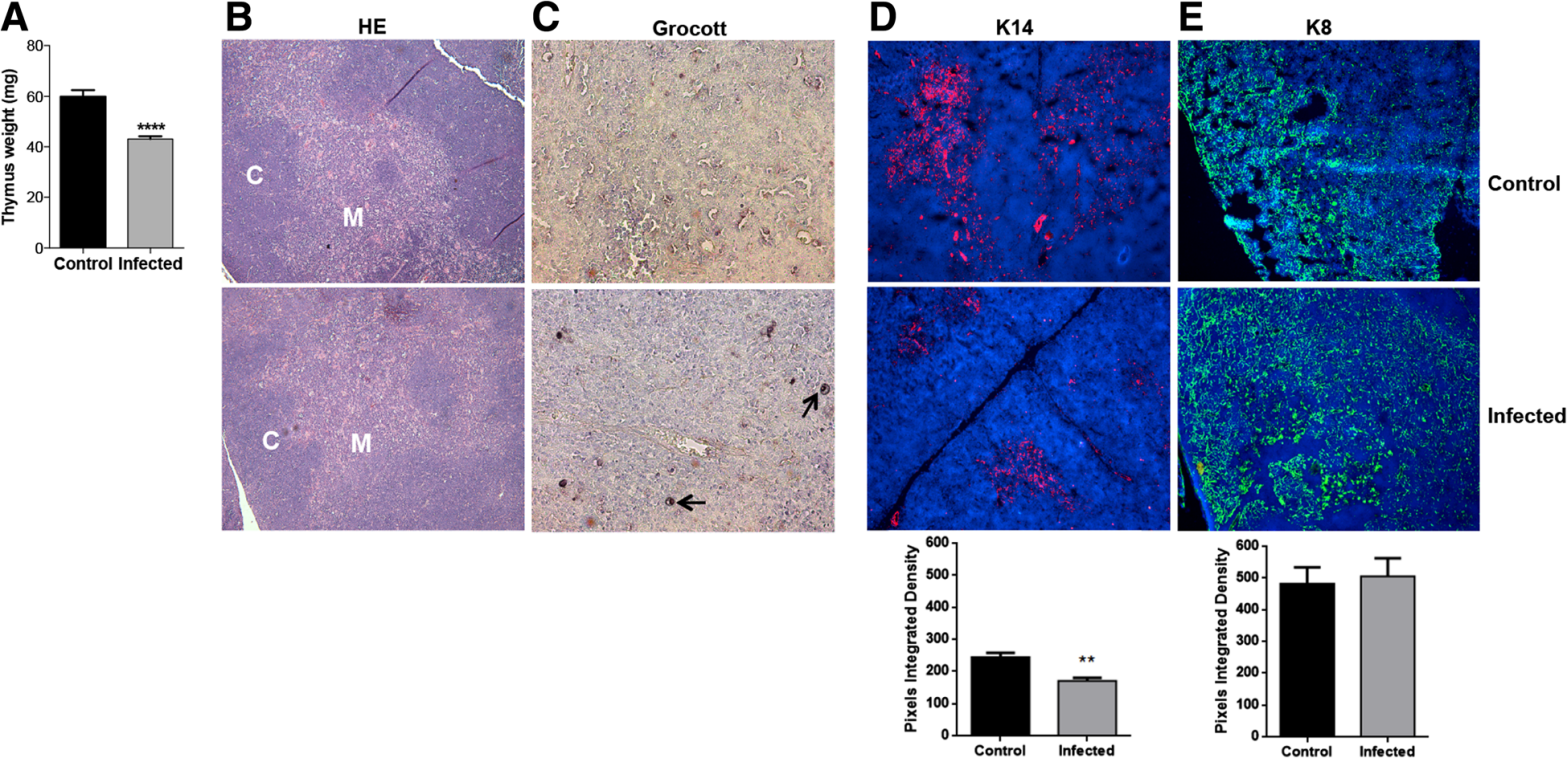
*P. brasiliensis* acute infection leads to thymic atrophy, alterations on thymus architecture and fungal invasion. Male Balb/c mice (*n* = 5 mice/group) were injected i.p. with Pb yeast cells. Seven days later, thymuses were analyzed for alterations on architecture and presence of yeast cells. (**a**) Loss of thymic weight following injection with yeast cells or PBS. (**b**) Cortico-medullary junction preserved by Hematoxylin-Eosin staining following injection with yeast cells or PBS. (**c**) Presence of yeast cells by Grocott staining method following injection with yeast cells or PBS, indicated by the arrows. (**d**) Unaltered expression of cytokeratin 14 by immunofluorescence following injection with yeast cells or PBS and (**e**) Diminished expression of cytokeratin 8 by immunofluorescence following injection with yeast cells or PBS. Photomicrography total magnification 100×. C-cortical and M-medullary areas. Data was analyzed by Student *t* test. Values of *p* ≤ 0, 01 (**) and *p* ≤ 0, 0001 (****) were considered statistically significant. Results are expressed by Mean ± SEM. Representative data from three independent experiments with similar results

To evaluate the effect of Pb infection on the thymus, male BALB/c mice were infected with 5x10^6^ yeast cells intraperitoneally. The animals were sacrificed seven days after infection and the thymuses were collected and weighted. The data obtained showed that the infection by Pb leads to significant reduction of the thymic weight in infected animals compared to control animals (Fig. 1a). We aimed to evaluate the possible structural changes in the thymus of mice infected with Pb. When the slides were stained for HE no structural changes between cortical and medullar region were observed (Fig. 1b) even though, viable fungal cells were detected by Grocott staining (Fig. 1c). Then we decided to investigate whether there was alteration in the expression and distribution of cortical and medullary epithelial cells by immunostaining of their cytokeratin 8 and 14, respectively. Thymuses of infected mice showed alterations in the arrangement of the medullar and cortical regions when compared with uninfected animals. These changes are evident when the immunostaining for cortical epithelial cells showed no significant alteration in the expression of cytokeratin but its spatial distribution appears to be misplaced with the medullar region (Fig. 1d). Meanwhile the analysis of the immunostaining for cytokeratin of medullar epithelial cells by integrated density shows that the expression of cytokeratin of medullar epithelial cells was significantly diminished (Fig. 1e).

### Genetic expression of the autoimmune regulator-AIRE and the cytokines IL-7, IL-2, IL-17 and TNFα are augmented in the thymuses of mice infected with P. brasiliensis

**Fig. 2:**
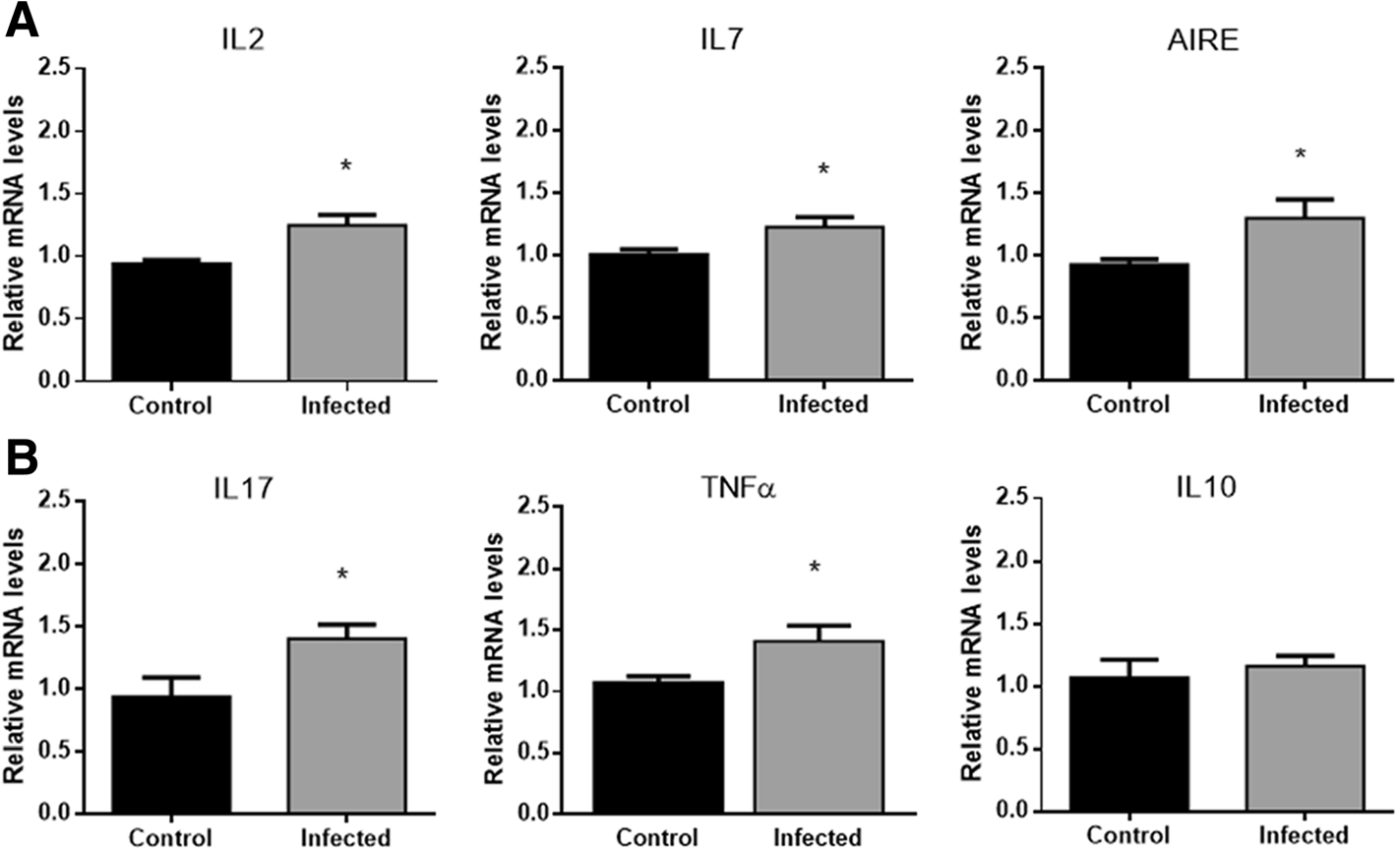
Gene expression of inflammatory mediators are augmented in the thymuses of mice infected with *P. brasiliensis*. Male Balb/c mice (*n* = 5 mice/group) were injected i.p. with Pb cells. Seven days later, thymuses were processed to mRNA extraction and analyzed for alterations on gene expression of AIRE, IL-7, IL-2, IL-17, IL-10 and TNFα, with GAPDH as housekeeping gene by real-time PCR. (**a**) Relative mRNA level of IL-2, IL-7 and AIRE in thymus of infected mice. (**b**) Relative mRNA level of IL-17, TNFα and IL-10. Expression levels of genes were represented as a relative copy numbers by using the method of delta threshold (2^-ΔΔCt^). Data was analyzed by Student *t* test. Values of *p* ≤ 0,05 (*) were considered statistically significant. Results are expressed by Mean ± SEM. Representative data from three independent experiments with similar results

In order to verify whether the gene expression of important cytokines and the transcription factor autoimmune regulator (AIRE) are altered in the thymus of infected mice, total mRNA was extracted from the thymuses of infected and non-infected mice and RT-PCR was performed to analyze the genetic expression of *IL*-*7*, *IL*-*2*, *IL*-*10*, *IL*-*17*, *TNF*α and *AIRE*. Results showed that the gene expression of IL-2, IL-7 and AIRE were significantly augmented in thymuses of Pb infected mice (Fig. 2a). Likewise gene expression of the inflammatory cytokines IL-17 and TNFα also showed higher levels in thymuses of Pb infected mice. The anti-inflammatory cytokine IL-10 had no altered levels in the thymuses of Pb infected mice (Fig. 2b).

### Chemokine signaling is altered in thymuses from infected mice and is correlated with increased migration of thymocytes

**Fig. 3:**
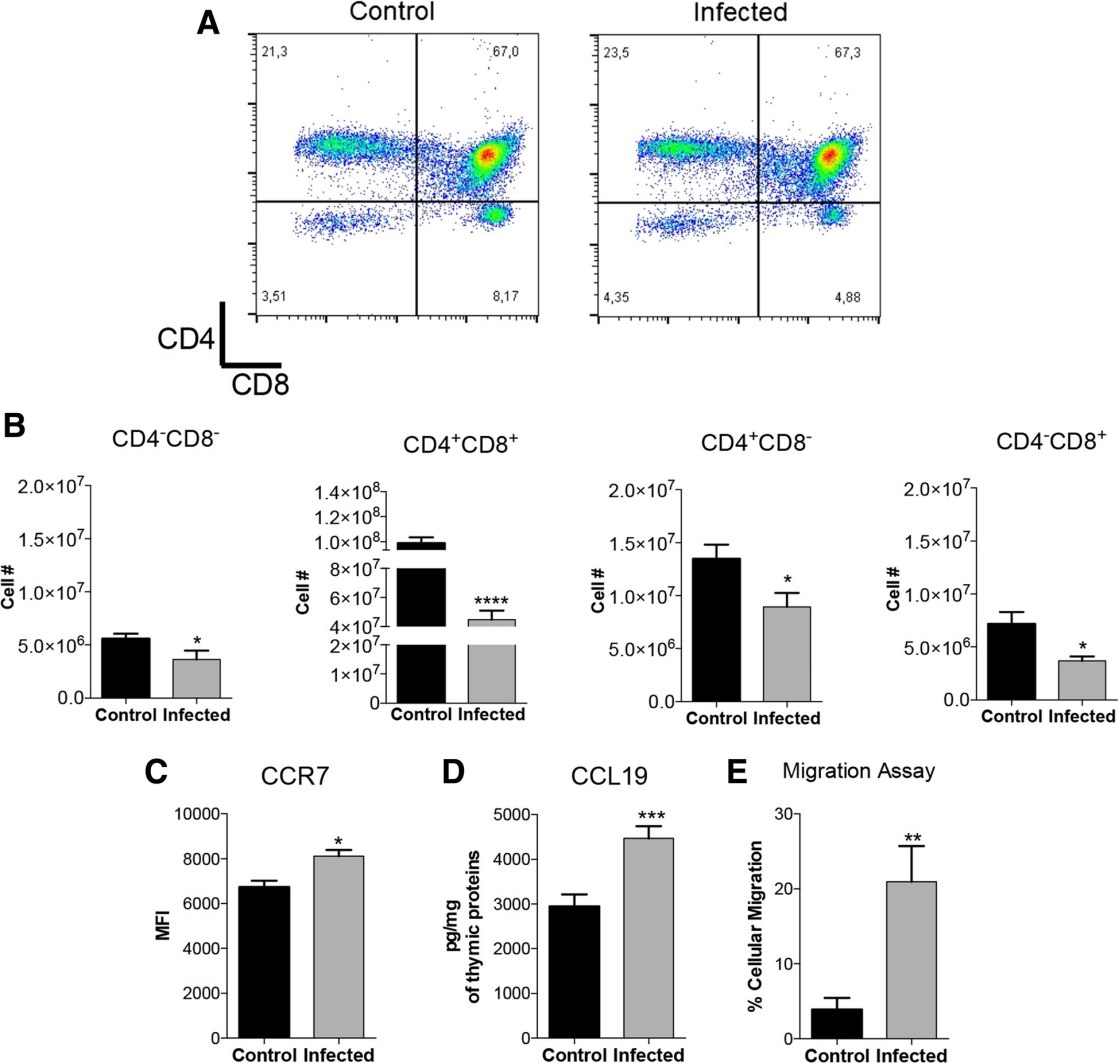
Increase of thymocytes migration due to increased protein expression of CCL19 and CCR7 in the thymuses of *P. brasiliensis* infected mice is correlated to altered thymocytes subpopulation. Male Balb/c mice (*n* = 5 mice/group) were injected i.p. with Pb cells. Seven days later, thymuses were both analyzed for trans-well migration assay, thymocytes subpopulation by flow cytometry and processed to total protein extraction, to analyze the expression of chemokine CCL19 and CCR7 in infected and control mice. (**a**) Frequency and (**b**) absolute number of thymocytes double positive and single positive subpopulation in infected mice. (**c**) Augmented expression of CCR7 and (**d**) CCL19 in thymus of infected mice. (**e**) Increased ex vivo thymocytes migration by Trans-well assay in infected mice. Data was analyzed by Student *t* test. Values of *p* ≤ 0,05 (*), *p* ≤ 0,001 (***), *p* ≤ 0,0001 (****) were considered statistically significant. Results are expressed by Mean ± SEM. Representative data from three independent experiments with similar results

The thymic involution and the histological changes observed in the presence of yeast in the thymuses from animals infected with Pb suggest that the thymus is a target organ also in PCM infection. The presence of the fungus in the thymus, an essential organ for maturation of lymphocytes and repertoire selection, may interfere with the differentiation process of thymocytes. Analysis of thymocytes subpopulations for CD4 and CD8 revealed that there were no changes in the relative frequencies of thymocytes subpopulations between healthy and infected mice (Fig. 3a), though there was a decrease in the absolute number of all thymocytes subpopulations at seven days of infection when compared to the control group (Fig. 3b). As the numbers of thymocytes subpopulations were altered, we evaluated whether there was an unbalanced chemokine expression caused by the Pb infection. To answer this question the expression of the chemokine ligand CCL19 and its receptor CCR7 was assessed. Both CCL19 and CCR7 expression were significantly increased in the thymuses from infected mice (Fig. 3c and Fig. d). Later, we evaluated the migration ability of thymocytes in Trans-well chambers. We incubated thymocytes from control and infected mice in the presence of CCL19. Results show that cells from Pb-infected mice presented a four-fold increased migration than to control cells (Fig. 3e).

### *Expression of* “*prohibited*” *TCRV*β *segments in T cells from spleens during acute* P. brasiliensis *infection*

**Fig. 4:**
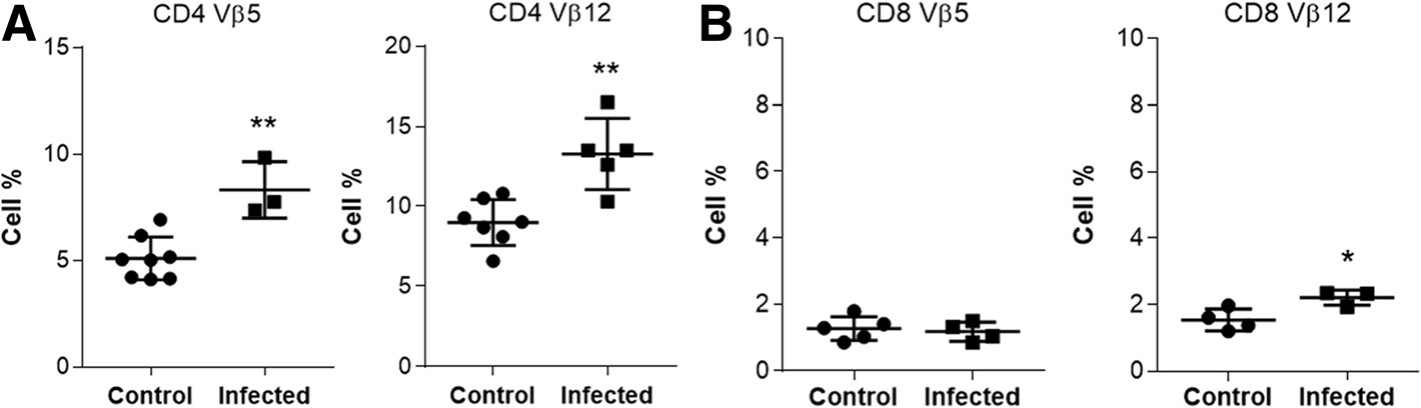
Expression of “prohibited” TCRVβ segments in T cells from spleens during acute *P. brasiliensis* infection. Male Balb/c mice (*n* = 5 mice/group) were injected i.p. with Pb cells. Seven days later, spleens were analyzed for expression of TCRVβ5 and Vβ12 segments in T cells from infected and control mice. Increased expression of TCRVβ5 and Vβ12 segments in (**a**) CD4 and TCRVβ12 segment in (**b**) CD8 T lymphocyte in spleens of infected mice. Data was analyzed by Student *t* test. Values of *p* ≤ 0,05 (*),*p* ≤ 0,01 (**) were considered statistically significant. Results are expressed by Mean ± SEM. Representative data from three independent experiments with similar results

During negative selection, thymocytes with high affinity for self-peptides/MHC undergo apoptosis, this process is crucial to eliminate T lymphocytes that could drive immune responses towards self-peptides in the periphery. In this context, we analyzed the percentage of T lymphocytes expressing prohibitive TCRVβ5 and Vβ12 segments. In normal conditions, there are low numbers of these cells in the thymus and also in the periphery of the immune system of BALB/c mice [36, 37]. Our results showed that spleens from infected mice presented an increased frequency of CD4^+^ and CD8^+^ T lymphocytes expressing TCRVβ5 and Vβ12 compared to controls (Fig. 4a and b). This observation suggests that the thymus is exporting T cells that should have been eliminated during the maturation process.

## Discussion

Paracoccidioidomycosis (PCM) is one of the most prevalent systemic mycosis in Latin America countries [38]. This disease affects mainly rural workers but immunosuppressed individuals can also develop it as an opportunistic disease [39–41]. *P. brasiliensis* is the causative agent of PCM. Pb has demonstrated tropism to organs of the immune system, as lymph nodes, spleen and the thymus. Although the thymus was considered as an immune privileged site, it is now consistent to say that antimicrobial responses take place in the thymus [42]. In this paper, we show that experimental infection with Pb yeast cells alters the thymic stroma with a decreased thymocytes cell subpopulation, increased thymocytes migration and higher expression of prohibited TCRVβ5 and Vβ12 segments in T lymphocytes from spleen.

The thymic microenvironment comprises a three-dimensional network of thymic epithelial cells (TECs), macrophages, dendritic cells, extracellular matrix components, enzymes and molecules, such as cytokines and chemokines in direct contact with thymocytes [43]. Local infection of the thymus and inflammatory mediators that follow systemic infection alter these thymic features and may impair the differentiation of pathogen-specific T cells, which may diminish host resistance to infection [42, 44, 45]. In this study, we demonstrated the ability of the strain Pb18 of *P. brasiliensis* to cause thymic atrophy while maintaining its viability in the thymic stroma at seven days after infection, in later time points of the infection more pathogens were found, including ones with budding yeasts, suggesting ability to thrive and multiply in the thymic microenvironment (*manuscript in preparation*). Even though the thymic weight in infected mice was 30 % lower than healthy mice, we observed no disruption in the cortico-medullary delimitation. We did observe that mTECs numbers were reduced in the medullar region of the thymus. Despite its role in the negative selection of SP thymocytes, mTECs are also involved in the maturation of SP thymocytes, which will be exported to the periphery as functional self-tolerant T cells [46]. We hypothesize that diminished presence of mTECs in Pb infected thymus might impair central tolerance and exportation of auto reactive T cells could be altered favoring the elimination of Pb-reactive T cells.

In order to evaluate these changes, we found that the presence of Pb in the thymus led to a higher gene expression of inflammatory cytokines genes such as TNFα and IL-17 causing a pro-inflammatory profile to control fungal dissemination. Interestingly, AIRE expression was found augmented in thymus from infected mice. We can only hypothesize that AIRE expression comprehends (a) an effort of the thymus to maintain negative selection in face of the reduction in mTEC numbers or (b) could be induced by the fungus to change the repertoire of T cells produced in the thymus and favor its survival. Unfortunately, literature lack concise data regarding both hypotheses.

Many studies have demonstrated that infection led to thymic atrophy by depletion of thymocytes subpopulation [9, 26–28, 44]. In our case, the loss of thymic weight was followed by: (a) diminished number of all four thymocytes subpopulations and (b) augmented thymocytes migration. The chemokine ligand CCL19 and its receptor CCR7 participate in thymocytes migration during the development of immature DP thymocytes to mature SP thymocytes throughout the thymus. Ueno et al. have shown that CCL19 is produced by mTECs and is located in the thymic medulla whereas CCR7 expression augments in cell surface of immature cortical DP thymocytes [47, 48]. Here we demonstrated that the expression of CCR7 and CCL19 were higher in the infected group than in control group. Our findings suggest that, during Pb infection, the thymocytes undergo maturation in an accelerated way. Actually, in *Plasmodium berghei*-infected mice we found that the thymus exports much more T cells than thymus from control mice (*manuscript in preparation*). Notwithstanding, T cell repertoire in *P. berghei*-infected mice was altered with the presence of prohibited-TCR-expressing T cells (*manuscript in preparation*). This altered T cell repertoire could lead to aggravation of autoimmune diseases. Indeed, we showed that following *P. berghei* infection, C57BL/6 mice develop a severe form of Experimental Autoimmune Encephalomyelitis, the mouse model of Multiple Sclerosis, by means of prematurely-egressed thymic Double-positive T cells [30]. Also, cells expressing prohibited TCRVβ segments were found in the lymph nodes of mice infected with *Trypanosoma cruzi*, probably as a result of the disbalance on thymic molecules, such as cytokines, chemokines and extracellular matrix elements that could promote the double-positive T cells escape [36, 49]. That could be the case of Pb infection. We found that spleen cells from Pb-infected mice contained a higher proportion of prohibited-TCR-expressing T cells than spleens cells from health mice.

## Conclusion

Taken together, our data show for the first time that, in acute Pb infection, the thymus is rendered atrophic as a consequence of elevated fungal burden, deregulated cytokine and chemokine expression, diminished absolute thymocytes subpopulations and enhanced migratory ability of thymocytes. In addition, as a possible consequence of thymic disarrangement, we observed an altered T cell repertoire in the periphery of the immune system. Further studies are required to ascertain the possible consequences of the thymic atrophy in experimental PCM.

### Ethics approval and consent to participate

This study was approved and carried out in accordance with the guidelines of the Institutional Committee for Use of Laboratory Animals (CEUA, # 2969–1).

### Consent for publication

Not applicable.

### Availability of data and materials

All the data supporting our findings is contained within the manuscript.

## Additional file

Additional file 1: The ARRIVE Guidelines Checklist. (PDF 1054 kb)

## Abbreviations

AIRE: autoimmune regulator
BSA: bovine serum albumin
CNS: central nervous system
Cy: cyanine
DN: double-negative
DP: double-positive
GAPDH: glyceraldehyde-3-phosphate dehydrogenase
HE: hematoxylin-eosin
HIV: human immunodeficiency virus
IL: interleukin
MHC: major histocompatibility complex
mRNA: messenger ribonucleic acid
Pb: paracoccidioides brasiliensis
PBS: phosphate-buffered saline
PCM: Paracoccidioidomycosis
SP: single-positive
TCR: T-cell receptor
TEC: thymic epithelial cell
Th cell: T helper cell
TNF: tumor necrosis factor
